# Impact of Western and Mediterranean Diets and Vitamin D on Muscle Fibers of Sedentary Rats

**DOI:** 10.3390/nu10020231

**Published:** 2018-02-17

**Authors:** Francesca Maria Trovato, Paola Castrogiovanni, Marta Anna Szychlinska, Francesco Purrello, Giuseppe Musumeci

**Affiliations:** 1 Department of Clinical and Experimental Medicine, University of Catania, Via S. Sofia, 78, 95123 Catania, Italy; trovatofrancesca@gmail.com (F.M.T.); fpurrell@unict.it (F.P.); 2 Department of Biomedical and Biotechnological Sciences, Human Anatomy and Histology Section, School of Medicine, University of Catania, Via S. Sofia, 87, 95123 Catania, Italy; pacastro@unict.it (P.C.); marta.sz@hotmail.it (M.A.S.)

**Keywords:** Mediterranean diet, high-fat diet, skeletal muscle, vitamin D, extra-virgin olive oil

## Abstract

Background: The metabolic syndrome is associated with sarcopenia. Decreased serum levels of Vitamin D (VitD) and insulin-like growth factor (IGF)-1 and their mutual relationship were also reported. We aimed to evaluate whether different dietary profiles, containing or not VitD, may exert different effects on muscle molecular morphology. Methods: Twenty-eight male rats were fed for 10 weeks in order to detect early defects induced by different dietary regimens: regular diet (R); regular diet with vitamin D supplementation (R-DS) and regular diet with vitamin D restriction (R-DR); high-fat butter-based diets (HFB-DS and HFB-DR) with 41% energy from fat; high-fat extra-virgin olive oil-based diets (HFEVO-DS and HFEVO-DR) with 41% energy from fat. IL-1β, insulin-like growth factor (IGF)1, Dickkopf-1 (DKK-1), and VitD-receptor (VDR) expressions were evaluated by immunohistochemistry. Muscle fiber perimeter was measured by histology and morphometric analysis. Results: The muscle fibers of the HEVO-DS rats were hypertrophic, comparable to those of the R-DS rats. An inverse correlation existed between the dietary fat content and the perimeter of the muscle fibers (*p* < 0.01). In the HFB-DR rats, the muscle fibers appeared hypotrophic with an increase of IL-1β and a dramatic decrease of IGF-1 expression. Conclusions: High-fat western diet could impair muscle metabolism and lay the ground for subsequent muscle damage. VitD associated with a Mediterranean diet showed trophic action on the muscle fibers.

## 1. Introduction

Muscle fiber number and size decrease with age, sedentary life, and chronic diseases, and this change is strictly related to reduced muscle function and strength [1]. The progressive loss of muscle mass leads to a condition called sarcopenia. Although undernutrition, impaired protein synthesis, and increased protein breakdown could lead to the onset of sarcopenia [2], an important issue in the Western world is the association of sarcopenia and obesity, or sarcopenic obesity, with a phenotype characterized by high adiposity and low lean body mass [3]. We are witnessing a great increase in obesity and metabolic syndrome which are quickly reaching a global epidemic scale, affecting over 500 million people worldwide [4]. This is critically associated with the development of cardio-metabolic disorders such as type-2 diabetes and non-alcoholic fatty liver disease (NAFLD) [5]. Dietary interventions, together with other important lifestyle changes, play a central role in achieving weight loss and modifying the natural history of these diseases [6,7]. The importance of vitamin D (VitD) and the impact of its deficiency on muscle health has been highlighted in several recent studies. These trophic properties are crucial in situations of loss of muscle mass, particularly in the context of chronic diseases [8]. Moreover, VitD deficiency is strictly related to sarcopenia, disability, and falls in the elderly [9,10]. Histological alterations of muscle fiber composition and diameter are associated with VitD deficiency [10]. Despite this evidence, the effectiveness of VitD administration in patients affected by muscle atrophy has not been assessed [11]. In obesity, the increase of circulating fatty acids leads to ectopic lipid deposition in other tissues, including skeletal muscle which represents the largest metabolically active tissue in the body (40% of body mass) [12]. Its maintenance is based on ongoing repair, regeneration, and growth [5]. In obese individuals, insulin resistance and mitochondrial dysfunction negatively impact muscle metabolism and physical performance [12]. Several authors reported that VitD deficiency is strongly associated with features of the metabolic syndrome and may play an important role in modifying the cardio-metabolic risk [13,14,15]. Low VitD levels were associated with higher risk of type-2 diabetes mellitus [14], and a study showed that VitD treatment improved insulin resistance among patients with baseline glucose intolerance, although other authors contradicted those observations [15]. Extra-virgin olive oil (EVO) is the principal fat and one of the cornerstones of the Mediterranean diet. The Mediterranean diet embodies the basics of healthy eating, coming from the traditional cooking style of countries bordering the Mediterranean Sea, including fruits, vegetables, fish, whole grains, red wine, and limited unhealthy fats [1,2]. Research has shown that the traditional Mediterranean diet reduces the risk of different chronic diseases [7]. The beneficial effects of EVO are ascribed to its phenolic compounds that exert beneficial effects interacting with the inflammatory cascade [16]. 

Our central suggestion is that the relationship between muscle and adipose tissue begins earlier than obesity and metabolic syndrome. 

The aim of the present study was to define whether different dietary regimens could influence skeletal muscle morphology in sedentary rats. We tried to mimic the dietary exposure to Western and Mediterranean diet by feeding rats with, respectively, a high-fat butter-based diet (HFB) and a high-fat extra-virgin olive oil-based diet (HFEVO), combined with VitD restriction (DR) or supplementation (DS). 

We hypothesized that high-fat butter-based diet could be detrimental for muscle trophism, whereas the same fat intake (41% of energy) could have beneficial effects when replacing the butter with EVO. The design included also the supplementation of VitD to evaluate if this nutrient could counteract or be synergic with those dietary profiles.

## 2. Materials and Methods

### 2.1. Breeding and Housing of Animals

Twenty-eight 7–9 weeks-old healthy Sprague/Dawley male rats (Envigo RMS S.r.l., Udine, Italy), with an average body weight of 271 ± 25 g, were housed in polycarbonate cages (cage dimensions: 10.25″ W × 18.75″ D × 8″ H) at controlled temperature (20–23 °C) and humidity during the whole period of the research, with free access to water and food and photoperiod of 12 h light/dark at the “Center for Advanced Preclinical In Vivo Research (CAPIR)”. Rats were allowed to adapt one week to their environment before the experiments began. Body weights, food and drink consumptions were monitored three days per week throughout the experiment. At the end of the experimental period (10 weeks), the animals were humanely sacrificed by exposure to a chamber filled with carbon dioxide until one minute after breathing stopped and then were decapitated. After euthanasia, skeletal muscle (anterior tibial of leg of right hind limb) was used to perform histological analyses and immunohistochemical evaluation. All procedures conformed to the guidelines of the Institutional Animal Care and Use Committee (I.A.C.U.C.) of the University of Catania (Protocol n. 2112015-PR of the 14.01.2015, Italian Ministry of Health). The experiments were conducted in accordance with the European Community Council Directive (86/609/EEC) and the Italian Animal Protection Law (116/1992). 

### 2.2. Experimental Design

Diets and origin of the extra-virgin olive oil: the extra-virgin olive oil (EVO) used for the experiment was a protected designation of origin (PDO) Monti Iblei Sottozona Gulfi (Oleificio Guccione di Divita Vito e G. SAS, Chiaramonte Gulfi, Sicily, Italy) obtained from the olive variety of Tonda Iblea. EVO was obtained by extraction through a continuous cold cycle and natural decantation. The acidity of the EVO was 0.18%. The composition of fatty acids was analyzed by an external chemical laboratory using Gas Chromatography (GC), High-Performance Liquid Chromatography (HPLC) (EN ISO 9936:2006), and Spectrophotometry. The composition of fatty acids in the EVO is reported in Table 1.

Seven different diets were used for the experiment, provided by Mucedola s.r.l. (Settimo Milanese, Milan, Italy) to which we sent the EVO for the specific preparation of rat chow. The composition of the experimental diets is reported in Table 2.

The 28 animals were divided into seven groups: R, control rats, fed with regular diet (*n* = 4); R-DS, rats fed with regular diet with vitamin D supplementation (4000 IU/Kg) (*n* = 4); R-DR, rats fed with common diet with vitamin D restriction (0 IU/Kg ) (*n* = 4); HFB-DS, rats fed with high-fat (butter) diet with vitamin D supplementation (4000 IU/Kg ) (*n* = 4); HFB-DR, rats fed with high-fat (butter) diet with vitamin D restriction (0 IU/Kg) (*n* = 4); HFEVO-DS, rats fed with high-fat (EVO) diet with vitamin D supplementation (4000 IU/Kg) (*n* = 4); HFEVO-DR, rats fed with high-fat (EVO) diet with vitamin D restriction (0 IU/Kg) (*n* = 4).

### 2.3. Histology

Skeletal muscle samples were fixed in 10% neutral buffered formalin (Bio-Optica, Milan, Italy), and, after overnight washing, were embedded in paraffin as previously described [17]. The samples were placed in the cassettes in longitudinal and cross directions after wax infiltration. Tissue samples (4–5 μm) were cut from paraffin blocks by a rotary manual microtome (Leica RM2235, Milan, Italy) and then mounted on silane-coated slides (Menzel-Gläser, Braunschweig, Germany) and preserved at room temperature. Afterwards, the sections were dewaxed in xylene, hydrated by graded ethanol, and stained by Hematoxylin and Eosin staining for histological evaluation, muscle fibers identification, detection of structural alterations, and histomorphometric measurements. The slides were examined with a Zeiss Axioplan light microscope (Carl Zeiss, Oberkochen, Germany), and pictures were taken with a digital camera (AxioCam MRc5, Carl Zeiss, Oberkochen, Germany).

### 2.4. Histomorphometric Analysis

Seven fields, the total area of which was about 150,000 µm^2^, randomly selected from each muscle (proximal area of anterior tibial of leg of right hind limb) cross section, were analyzed for morphometric analysis. The perimeter of the muscle fibers was considered and calculated using a software for image acquisition (AxioVision Release 4.8.2-SP2 Software, Carl Zeiss Microscopy GmbH, Jena, Germany). Negative images were used for a better software performance in the morphometric analysis. The data were expressed as mean ± standard deviation (SD). The statistical significance of the results was then evaluated. A Zeiss Axioplan light microscope (Carl Zeiss, Oberkochen, Germany) fitted with a digital camera (AxioCam MRc5, Carl Zeiss, Oberkochen, Germany) was used to take digital micrographs; the evaluations were made by three blinded investigators, whose evaluations were assumed to be correct if the recorded values were not significantly different. In case of dispute concerning interpretation, the case was reconsidered to reach a unanimous agreement [18]. 

### 2.5. Immunohistochemistry (IHC)

Skeletal muscle samples were processed for immunohistochemical analysis as previously described [19]. In detail, the slides were dewaxed in xylene, hydrated by graded ethanol, incubated for 30 min in 0.3% hydroperoxyl (HO_2_)/methanol to remove endogenous peroxidase activity and then rinsed in phosphate-buffered saline (PBS; Bio-Optica, Milan, Italy) for 20 min. In order to unmask the antigenic sites, the samples were stored in capped polypropylene slide holders with citrate buffer (10 mM citric acid, 0.05% Tween 20, pH 6.0; Bio-Optica, Milan, Italy) and heated for 5 min for three times through a microwave oven (750 W, LG Electronics Italia S.p.A., Milan, Italy). In order to prevent non-specific binding of the antibodied, a blocking step with 5% bovine serum albumin (BSA, Sigma, Milan, Italy) in PBS for 1 h in a moist chamber was performed before the application of the primary antibodies. The sections were then incubated overnight at 4 °C with the following antibodies: rat monoclonal anti-vitamin D receptor (ab115495; Abcam, Cambridge, UK), work dilution in PBS (Bio-Optica, Milan, Italy) 10 μg/mL; rabbit polyclonal anti-IL-1β (ab2105; Abcam, Cambridge, UK), diluted 1/100 in PBS (Bio-Optica, Milan, Italy); goat polyclonal anti-insulin-like growth factor (IGF)-1 (sc-7144; Santa Cruz Biotecnology, Inc., Dallas, Texas, U.S.A), diluted 1/100 in PBS (Bio-Optica, Milan, Italy), and rabbit polyclonal anti-Dickkopf-1 (DKK-1) (sc-25516; Santa Cruz Biotecnology, Inc., Dallas, TX, U.S.A), diluted 1/100 in PBS (Bio-Optica, Milan, Italy). The samples were then coated with a biotinylated antibody (horseradish peroxidase (HRP)-conjugated anti-goat and anti-rabbit were used as secondary antibodies), and the immune complexes were detected with peroxidase-labeled streptavidin (labeled streptavidin-biotin (LSAB) + System-HRP, K0690, Dako, Glostrup, Denmark), after incubation for 10 min at room temperature. The immunoreaction was detected by incubating the sections for 2 min in a 0.1% 3,3′-diaminobenzidine, 0.02% hydrogen peroxide solution (DAB substrate Chromogen System; Dako, Denmark). The slides were lightly counterstained with Mayer’s Hematoxylin (Histolab Products AB, Goteborg, Sweden) and mounted in GVA mount (Zymed, Laboratories Inc., San Francisco, CA, USA). 

### 2.6. Computerized Densitometric Measurements and Image Analysis

An image analysis software (AxioVision Release 4.8.2-SP2 Software, Carl Zeiss Microscopy GmbH, Jena, Germany), which quantifies the level of staining of positive anti-collagen I, anti-IL-1β, anti-vitamin D receptor, anti-IGF-1, anti-DKK-1 antibodies immunolabelling, was used to calculate the densitometric count (pixel2) (immunolabelling intensity) and the percentage of the immunostained area (immunolabelling extension) in seven fields, the area of which was about 150,000 µm^2^, randomly selected from each muscle (proximal area of anterior tibial of leg of right hind limb) cross section. Digital micrographs were taken using the Zeiss Axioplan light microscope (Carl Zeiss, Oberkochen, Germany), using a lens with a magnification of × 20, i.e., total magnification 200) fitted with a digital camera (AxioCam MRc5, Carl Zeiss, Oberkochen, Germany). Three blinded investigators (two anatomical morphologists and one histologist) made the evaluations that were assumed to be correct if the recorded values had no statistically significant difference [20]. If disputes concerning interpretation occurred, a unanimous agreement was reached after sample re-evaluation [21].

### 2.7. Statistical Analysis

The statistical analysis was performed using GraphPad Instat^®^ Biostatistics version 3.0 software (GraphPad Software, Inc., La Jolla, CA, USA) and IBM SPSS Statistics (version 20, IBM corporation, Somers, Armonk, NY, USA) [20,21]. The analysis of variance (ANOVA)-Tukey’s multiple comparisons test was used for comparisons between more than two groups. The correlations between all variables were tested by Pearson’s correlation coefficient. A *p*-value of less than 0.05 (*p* < 0.05) was considered statistically significant; *p*-values of less than 0.01 (*p* < 0.01) were considered highly statistically significant. The data are presented as the mean ± SD. 

## 3. Results

### 3.1. Body Weight and Food Intake

**Figure 1:**
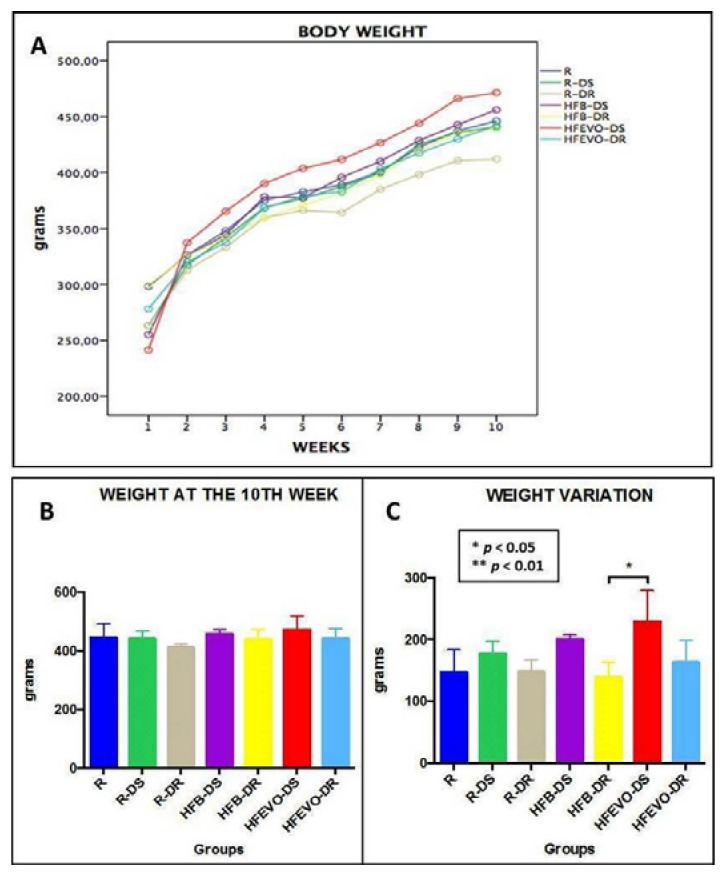
Graphs: (**A**) and (**B**): body weight over 10 weeks, the differences between groups were not significant (*p* > 0.05). (**C**): body weight variation over 10 weeks, a slight trend toward a greater weight gain in the DS experimental groups is evident; the only statistically significant difference was between HFB-DR and HFEVO-DS (*p* < 0.05). R: Regular diet; R-DS: Regular diet with vitamin D supplementation; R-DR: Regular diet with vitamin D restriction; HFB-DS: High-fat butter-based diet with vitamin D supplementation; HFB-DR: High-fat butter-based diet with vitamin D restriction; HFEVO-DS: High-fat extra-virgin olive oil-based diet with vitamin D supplementation; HFEVO-DR: High-fat EVO-based diet with vitamin D restriction.

Body weights and food and drink consumptions were monitored for all groups, three days per week throughout the experiment. In relation to body weight (Figure 1A), we observed a physiological increase in all groups, but the differences between groups at the end of the experiment (10th week) were not significant (*p* > 0.05) (Figure 1B). When analyzing the weight variation, a slight trend toward a greater weight gain was seen in the DS experimental groups in comparison to the corresponding DR groups, without reaching significance. The only statistically significant difference was between HFB-DR and HFEVO-DS (*p* < 0.05) (Figure 1C). Also, the food consumptions did not show significant differences between groups (*p* > 0.05) (data not shown).

### 3.2. Histology

Hematoxylin & Eosin staining was performed to detect structural alterations in the muscle tissue of the experimental groups. None of the groups showed damage to the histological structure of the muscle fibers. However, muscle fiber hypertrophy was observed in groups R-DS and HFEVO-DS, and hypotrophy in the HFB-DR group, as better reported in the histomorphometric analysis of muscle fibers.

### 3.3. Histomorphometric Analyses

**Figure 2:**
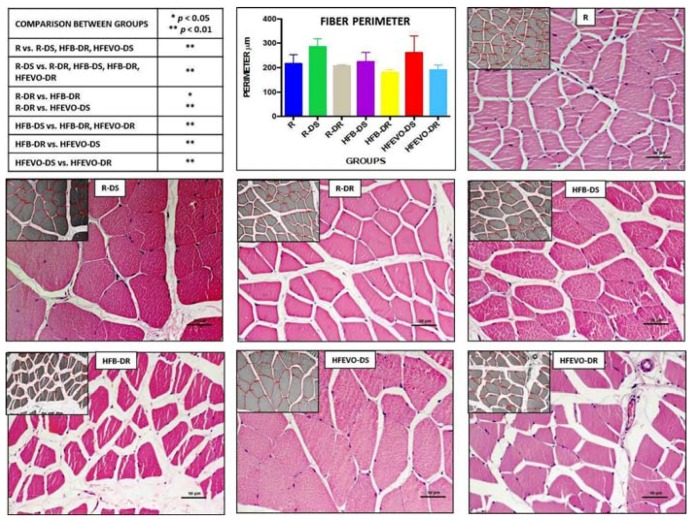
Hematoxylin & Eosin staining. Image analysis by software with morphometric analysis of the perimeter (μm) of the muscle fibers (inserts) and a graph representing the mean values of the perimeter (μm) in each group with statistical analysis (*p*-values in the table). For details, see the text. The data are presented as mean ± SD. Scale bars: 50 µm.

In the morphometric analysis of the perimeter (μm) (mean ± SD) of the muscle fibers, the comparison between group R (health control) versus all other groups highlighted a statistically highly significant hypertrophy in groups R-DS and HFEVO-DS (*p* < 0.01) and a statistically highly significant hypotrophy in group HFB-DR (*p* < 0.01). In detail: R vs. R-DS, HFB-DR, HFEVO-DS had *p* < 0.01; R-DS vs. R-DR, HFB-DS, HFB-DR, HFEVO-DR had *p* < 0.01; R-DR vs. HFB-DR, HFEVO-DS had, respectively, *p* < 0.05 and *p* < 0.01; HFB-DS vs. HFB-DR, HFEVO-DR had *p* < 0.01; HFB-DR vs. HFEVO-DS had *p* < 0.01; HFEVO-DS vs. HFEVO-DR had *p* < 0.01 (Figure 2). Further analyses and comparisons between the groups are reported in the paragraph “Statistical analysis of the histomorphometric results”.

### 3.4. Statistical Analysis of the Histomorphometric Results

The fiber perimeters correlated positively with the dietary VitD content (*r* = 0.603; *p* < 0.001) and inversely with the dietary fat content (*r* = −0.222; *p* < 0.05). In our model, weight had no correlation with muscle fiber perimeter (*r* = 0.003). A multiple linear regression was calculated to predict muscle fiber perimeter in relation to weight at the end of the experiment, VitD, and fat content in diet. The results of the multiple linear regression indicated that there was a collective significant relationship between the fiber perimeter, VitD, and dietary fat, (*F* = 34.827; *p* < 0.001, *r*^2^ = 363). The individual predictors were examined further, and indicated that dietary VitD (*t* = 5.901; *p* < 0.001) and dietary fat (*t* = −2.609; *p* < 0.05) were significant predictors in the model.

### 3.5. Immunohistochemistry (IHC) Observations

#### 3.5.1. Interleukin (IL)-1β

**Figure 3:**
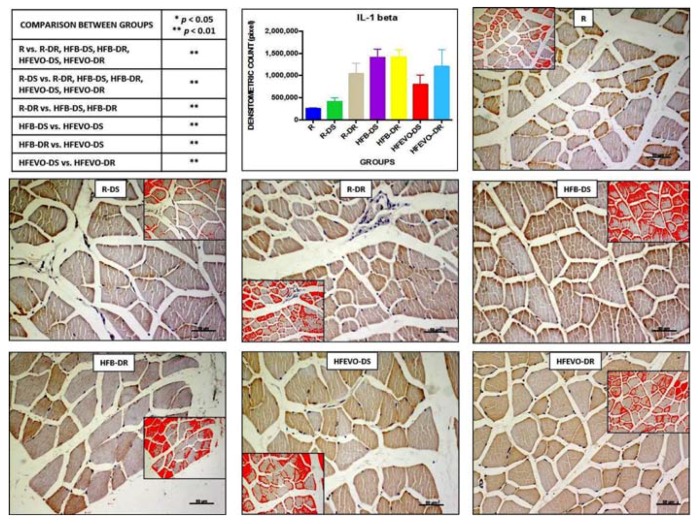
IL-1β immunostaining, image analysis by software in which the red color represents the immunolabelling (inserts), and a graph representing the immunostained area % with statistical analysis (*p*-values in the table). For details, see the text. The data are presented as mean ± SD. Scale bars: 50 µm.

IL-1β immunostaining in muscle fibers was mainly membranous and cytoplasmic and rarely nuclear; sometimes, it was detectable in the muscle satellite cells. The intensity of IL-1β immunostaining (densitometric count pixel2) was detected in many fields of the analyzed samples, albeit at different levels. In detail: the immunostaining in R was lower than in R-DR, HFB-DS, HFB-DR, HFEVO-DS, HFEVO-DR (*p* < 0.01); in R-DS, it was lower than in R-DR, HFB-DS, HFB-DR, HFEVO-DS, HFEVO-DR (*p* < 0.01); in R-DR, it was lower than in HFB-DS, HFB-DR (*p* < 0.01); in HFB-DS, it was higher than in HFEVO-DS (*p* < 0.01); in HFB-DR, it was higher than in HFEVO-DS (*p* < 0.01); in HFEVO-DS, it was lower than in HFEVO-DR (*p* < 0.01) (Figure 3). In relation to the immunostained area %, the statistical results were analogues to those of the intensity of immunostaining (data not shown).

#### 3.5.2. IGF-1

**Figure 4:**
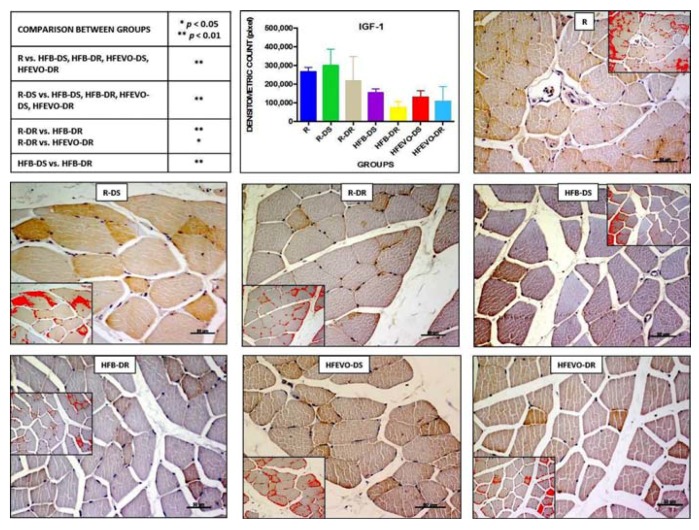
IGF-1 immunostaining, image analysis by software in which the red color represents the immunolabelling (inserts), and a graph representing the intensity of immunostaining (densitometric count pixel2) with statistical analysis (*p*-values in the table). For details, see the text. The data are presented as mean ± SD. Scale bars: 50 µm.

In muscle tissue, IGF-1 immunostaining was mainly membranous and cytoplasmic and rarely nuclear. The intensity of IGF-1-immunostaining (densitometric count pixel2) was detected at different degrees in all groups. In detail: in R, the immunostaining was higher than in HFB-DS, HFB-DR, HFEVO-DS, HFEVO-DR (*p* < 0.01); in R-DS, it was higher than in HFB-DS, HFB-DR, HFEVO-DS, HFEVO-DR (*p* < 0.01); in R-DR, it was higher than in HFB-DR (*p* < 0.01); in R-DR, it was higher than in HFEVO-DR (*p* < 0.05); in HFB-DS, it was higher than in HFB-DR (*p* < 0.01) (Figure 4). With regard to the immunostained area %, it showed a lesser extension with respect to IL-1β, and the statistical results were analogues to those of the intensity of IGF-1 immunostaining (data not shown).

#### 3.5.3. Dickkopf (DKK) Wingless-type (WNT) Signaling Pathway Inhibitor 1

**Figure 5:**
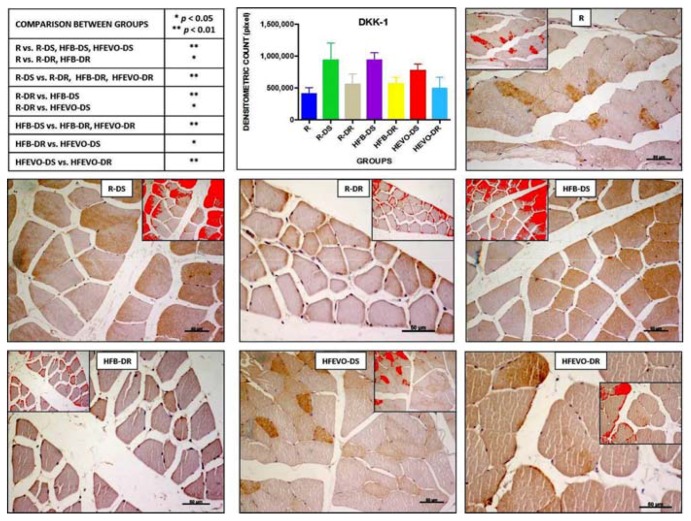
DKK-1 immunostaining, image analysis by software in which the red color represents the immunolabelling (inserts), and a graph representing the intensity of immunostaining (densitometric count pixel2) with statistical analysis (*p*-values in the table). For details, see the text. The data are presented as mean ± SD. Scale bars: 50 µm.

DKK-1-immunostaining was mainly membranous and cytoplasmic and rarely nuclear in the muscle fibers. The intensity of DKK-1-immunostaining (densitometric count pixel2) was detected in all groups at different levels. In detail: the immunostaining in R was lower than in R-DS, HFB-DS, HFEVO-DS (*p* < 0.01); in R, it was lower than in R-DR, HFB-DR (*p* < 0.05); in R-DS, it was higher than in R-DR, HFB-DR, HFEVO-DR (*p* < 0.01); in R-DR, it was lower than in HFB-DS (*p* < 0.01) and HFEVO-DS (*p* < 0.05); in HFB-DS, it was higher than in HFB-DR and HFEVO-DR (*p* < 0.01); in HFB-DR, it was lower than in HFEVO-DS (*p* < 0.05); in HFEVO-DS, it was higher than in HFEVO-DR (*p* < 0.01) (Figure 5). DKK-1 immunostaining was highlighted in the sarcoplasm in groups in which it had a greater extension (immunostained area %), whereas it was mainly highlighted close to the plasma membrane in groups where the immunostained area % had a smaller extension. The statistical results of the immunostained area % were analogues to those of the intensity of immunostaining (data not shown).

#### 3.5.4. VDR

**Figure 6:**
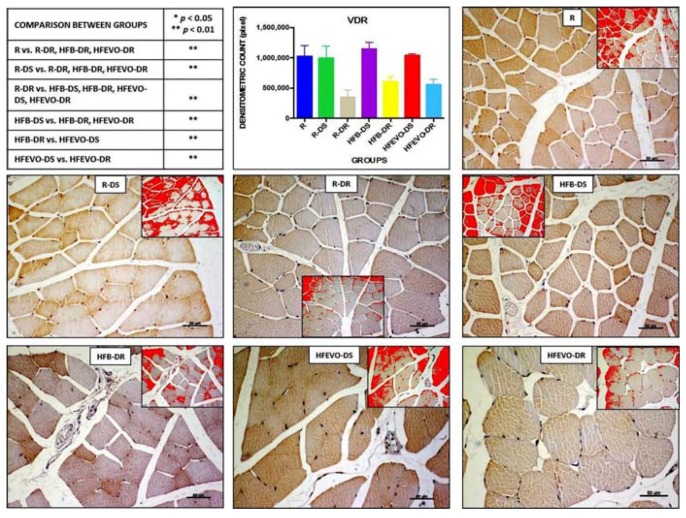
VDR immunostaining, image analysis by software in which the red color represents the immunolabelling (inserts), and a graph representing the intensity of immunostaining (densitometric count pixel2) with statistical analysis (*p*-values in the table). For details, see the text. The data are presented as mean ± SD. Scale bars: 50 µm.

In muscle fibers, VDR immunostaining was mainly cytoplasmic and, in some samples, nuclear. The intensity of VDR immunostaining (densitometric count-pixel2) was higher in R, R-DS, HFB-DS, and HFEVO-DS groups. In detail: in R, the immunostaining was higher than in R-DR, HFB-DR, HFEVO-DR (*p* < 0.01); in R-DS, it was higher than in R-DR, HFB-DR, HFEVO-DR (*p* < 0.01); in R-DR, it was lower than in HFB-DS, HFB-DR, HFEVO-DS, HFEVO-DR (*p* < 0.01); in HFB-DS, it was higher than in HFB-DR, HFEVO-DR (*p* < 0.01); in HFB-DR, it was lower than in HFEVO-DS (*p* < 0.01); in HFEVO-DS, it was higher than in HFEVO-DR (*p* < 0.01) (Figure 6). In relation to the immunostained area %, the statistical results were analogues to those of the intensity of VDR immunostaining (data not shown).

### 3.6. Statistical Analysis of Immunohistochemistry

IL-1β negatively correlated with muscle fiber perimeter (*r* = −0.420; *p* < 0.01), VDR (*r* = 0.322; *p* < 0.05), and dietary VitD (*r* = −0.274; *p* < 0.05), while it was positively related to dietary fat content (*r* = 0.674; *p* < 0.01). VDR was positively correlated with dietary VItD content (*r* = 0.799; *p* < 0.01), weight (*r* = 0.327; *p* < 0.01), fiber perimeter (*r* = 0.410; *p* < 0.01), and DKK-1 (*r* = 0.445; *p* < 0.01). DKK-1 was positively related to dietary VItD content (*r* = 0.697; *p* < 0.01), VDR, and fiber perimeter (*r* = 0.493; *p* < 0.01). A multiple linear regression was calculated to predict muscle fiber perimeter based on IL-1β, VDR, DKK-1. The results of the multiple linear regression indicated that there was a collective significant relationship between the fiber perimeter, IL-1β, and DKK-1. About 41.6% of the variance is explained by the model (*r*^2^ = 0.416; *F* = 21.330; *p* < 0.001). The individual predictors were examined further and indicated that DKK-1 (*t* = 4.95; *p* < 0.001) and IL-1β (*t* = −4.20; *p* < 0.001) were significant predictors in the model.

## 4. Discussion

Several studies have reported that a Mediterranean dietary regimen has a favorable role in the prevention of sarcopenia [22,23]. Moreover, in our previous study, we demonstrated that EVO possesses anti-oxidative properties and can improve the adaptive response of the body in conditions of oxidative stress [24,25], thanks to its ability to scavenge free radicals, thus preventing cellular injury. Phenolic compounds in EVO have been also shown to exert beneficial effects on oxidative stress with subsequent positive effect on disease risk. Our results confirmed the original hypothesis, since the muscle fibers of rats fed with EVO-based high-fat diet (HEVO-DS) were hypertrophic, comparable to those of the regular diet (R-DS) group, confirming that EVO does not have the same detrimental effect on muscle fiber as other kinds of fat, in particular butter, as shown in HFB groups. In fact, we found an inverse correlation between the dietary fat content and the perimeter of muscle fibers (*p* < 0.01). In HFB-DR, the muscle fibers appeared hypotrophic, probably because VitD action was lacking and the HFB diet led to the increase of IL-1β expression and to a dramatic decrease of IGF-1 expression. When the supplementation of VitD antagonized the fat effect, as in HFB-DS, the fibers were not different from the control. VitD depletion, per se, did not show any effects when associated with the three dietary profiles, and this was probably due to the relatively short term of the restriction in young rats. Conversely, EVO did not show detrimental effects on muscle fiber size in the HFEVO-DR group, although IL-1β was more expressed in comparison to control. Our results are in line with the recent literature. Obesity is a known state of chronic inflammation associated with a reduction in skeletal muscle regeneration [5]. Indeed, obese mice displayed abnormal muscle fiber size, providing evidence for a cross-talk between human obese adipocytes and muscle cells, leading to muscle atrophy [26]. Such studies on obese mice [26] found the same results that we highlighted in our high-fat diet model, in which rats are not obese. Lipids accumulate ectopically in non-adipose tissues like skeletal muscles, impairing the repair process following injury. Considerable interest was raised in exploring the putative pleiotropic functions of VitD. VitD exerts its biological effects [27] via binding to VitD receptor (VDR) and stimulates muscle recovery [28]. Moreover, acute inflammation triggers the process of muscle regeneration [29], with a critical role of the immune cells. Several observational studies have been performed on the role of VitD in skeletal muscle proliferation, differentiation, and apoptosis as well as in immune cells regulation [30]. VDR knock-out mice have provided evidence for a role of VDR in the development and differentiation of skeletal muscle, showing smaller myofibers in knock-out mice than in wild-type mice [31]. Animal models suggested that the administration of VitD leads to an increase in proliferation and a decrease in apoptosis in injured muscle [32], and, conversely, a high-fat diet with VitD deficiency would markedly impair bone and muscle metabolism [33]. A clinical trial highlighted that VitD supplementation in sedentary women increased VDR expression and muscle fiber size [34]. These results indicate a direct effect of VitD on myocites, leading to the enhancement of skeletal muscle recovery [35]. VitD has effects not only on muscle cells [28], but also on immune cell functions [36], so we decided to investigate the relationship between VitD and inflammation. Several recent studies have identified inflammatory mediators, such as IL-1, IL-6, and Tumor Necrosis Factor (TNF)-α, as contributing to the development of sarcopenia [37], since they may induce muscle atrophy promoting protein degradation and reactive oxygen species (ROS) accumulation [38,39]. Adipocytes secrete inflammatory cytokines as well as adipokines, leptin, and adiponectin, which promote inflammation and fat mass accumulation, impairing muscle mass formation [38]. IL-1β is a major cytokine produced largely by adipose tissue macrophages. Its release is enhanced in obesity [40] and it is implicated in the development of insulin resistance [40]. The blood concentration of anti-inflammatory cytokines IL-10 and IL-13 were found decreased in VitD-deficient subjects following physical training, compared to controls. Conversely, in a rat model, VitD supplementation modulated pro-inflammatory cytokine expression in muscle following intensive physical exercise [41]. Moreover, a recent research by our group demonstrated similar effects of the administration of EVO with diet on muscles undergoing exhaustive exercise in rats [25], supporting the view that EVO can improve the adaptive response of the body in conditions of oxidative stress [25]. In the present study, indeed, IL-1β was more expressed in HFB groups, but in regular diet and HFEVO groups it was clear that the VitD restriction led to a higher expression of IL-1β, confirming the hypothesis that VitD could be beneficial against inflammation. Furthermore, in comparison to butter, EVO is rich in monounsaturated fatty acids (MUFA) such as oleic acid, that exert antinflammatory activity [41,42]. Conversely, the concentration of polyunsaturated fatty acids (PUFA) in HFEVO is lower than in regular diet, and this can further explain the lower expression of IL-1β in the latter. 

Insulin resistance has been associated to cases of both excess fat and sarcopenia, given that skeletal muscle is one of the major target tissues of insulin action [43]. Growth hormone (GH) and insulin-like growth factor-1 (IGF-1) act on body composition, protein metabolism in the skeletal muscle, as well as bone growth and remodeling [44] and lipid and glucose homeostasis. At the molecular level, IL-6 and IL-1β downregulate IGF signaling, decreasing muscle protein synthesis [45]. Fatty liver has been related to the reduction of insulin sensitivity at the level of skeletal muscle and adipose tissue [46]. In fact, the main source of circulating IGF-1 is exactly the liver, which is considered a target tissue for VitD [47]. Authors showed that VitD supplementation increased circulating IGF-1 [48], and we confirmed this observation, but what was clearer is the reduction of IGF-1 in both high-fat diets. Similarly, muscle size and strength, as well as IGF-1 levels decreased in mice with diet-induced NAFLD, and, as in our study, the morphological aspects of sarcopenia were observed in early stages, prior to the development of liver fibrosis [49]. Another pathway we tried to explore in order to explain how VitD could influence muscle is the Wnt (Wingless-type MMTV (mouse mammary tumor virus) integration site family)/β-catenin pathway. In fact, the targets for VitD include low-density lipoprotein receptor-related protein (LRP)5, the Wnt coreceptor, that plays a key role in osteoblast proliferation, differentiation, and function [50]. A new field of research linking VitD with cell proliferation and Wnt pathway is oncology. In breast and colon cancer, VitD increased dose dependently the expression of the extracellular canonical Wnt inhibitor, DKK-1, that is associated with growth inhibition, showing a protective role of VitD against breast cancer development, progression, and metastasis [51]. From this consideration, we hypothesized that VitD could exert its action in muscle through the expression of DKK-1. The latter, indeed, is involved in many processes of bone metabolism, but exerts also action on muscle. This is well known in cardiomyocytes, where Wnt/β-catenin signaling has been implicated in the regulation of cardiac remodeling and injury responses [52]. Wnt/β-catenin signaling promotes fibrosis in response to injury in order to prevent cardiac dilation [52] and, interestingly, it has also been reported in fibrotic diseases of other organs (liver, lung, and kidney), since it is crucial for the differentiation of fibroblasts and for collagen production [52]. Increased Wnt signaling may inhibit myogenicity, impairing the muscle regenerative potential by promoting the transition of aged skeletal muscle to fibrogenic tissue, thereby accelerating aging [38]. This lineage conversion can be suppressed by Wnt inhibitors [53], like DKK-1 that is upregulated by VitD [54]. In our study, DKK-1 expression correlated directly with muscle fiber perimeter and VDR expression and, most importantly, with dietary VitD supplementation, without differences attributable to different fats. Thus, by comparing the different dietary profiles, we can conclude that VitD supplementation causes muscle fiber hypertrophy both in regular and HFEVO diet, probably through a pathway involving IGF-1 and DKK-1. 

Our results need to be strengthened by further studies, since the small sample size was a major limitation.

## 5. Conclusions

Our morphological results are consistent with the original hypothesis of the study and show the effect of nutrition on skeletal muscle as an emerging topic of interest. High-fat western diet could impair muscle metabolism and create a basis for subsequent muscle damage. Vitamin D shows trophic action on muscle fibers, not only in rats fed with regular diet, but also in the case of a diet mimicking the Mediterranean diet. Our research supports the hypotheses that the relationship between muscle and adipose tissue starts earlier than obesity and that we can modify muscle metabolism with a dietary intervention. However, this is a preliminary research, and further study is needed to strengthen and confirm our data.

## Figures and Tables

**Table 1:** Composition % m/m of fatty acids in extra-virgin olive oil (EVO).

Fatty Acids	EVO
Palmitic acid 16:0	14.41
Palmitoleic acid 16:1	1.31
Stearic acid 18:0	2.18
Oleic acid 18:1	70.38
Linoleic acid 18:2	9.69
Linolenic acid 18:3	0.84

**Table 2:** Composition of the experimental diets.

Compound	Regular (R) 9.0% of Energy from Fat	High-Fat Butter (HFB) 41% of Energy from Fat	High-Fat EVO (HFEVO) 41% of Energy from Fat
Water (% *w*/*w*)	10.69	8.54	8.50
Protein (% m/m)	22.90	21.08	21.03
Fat (% m/m)	3.54	21.05	21.16
Fiber (% m/m)	3.63	3.23	3.23
Ash (% m/m)	7.55	7.26	7.26
NFE (% m/m)	51.44	38.58	38.58
Carbohydrates (% m/m)	55.07	41.81	41.81
M.E. (kcal/kg)	2757	3801	3801
Palmitic acid 16:0 (mg/kg)	6127	46,149	30,470
Palmitoleic acid 16:1 (mg/kg)	308	2170	2771
Stearic acid 18:0 (mg/kg)	1336	20,139	4612
Oleic acid 18:1 (mg/kg)	8638	42,976	148,924
Linoleic acid 18:2 (mg/kg)	17,300	12,845	20,504
Linolenic acid 18:3 (mg/kg)	2072	1672	1777
DIET	Vitamin D		
R	1400 IU/kg		
R-DS HFB-DS HFEVO-DS	4000 IU/kg		
R-DR HFB-DR HFEVO-DR	0 IU/kg		

NFE: nitrogen-free extract; M.E.: Metabolizable Energy; R: Regular diet; R-DS: Regular diet with vitamin D supplementation; R-DR: Regular diet with vitamin D restriction; HFB-DS: High-fat butter-based diet with vitamin D supplementation; HFB-DR: High-fat butter-based diet with vitamin D restriction; HFEVO-DS: High-fat EVO-based diet with vitamin D supplementation; HFEVO-DR: High-fat EVO-based diet with vitamin D restriction.
